# Rescue Veno-Venous Extracorporeal Membrane Oxygenation for Aspiration-Induced Refractory Hypoxaemia Before Non-Deferrable Abdominal Surgery: A Case Report

**DOI:** 10.3390/jcm15176746

**Published:** 2026-08-30

**Authors:** Simon Dolenc, Primož Kastelic, Anja Kramarič Lozar, Primož Gradišek

**Affiliations:** 1 Clinical Department of Anaesthesiology and Intensive Therapy, University Medical Centre Ljubljana, 1000 Ljubljana, Slovenia; 2 Faculty of Medicine, University of Ljubljana, 1000 Ljubljana, Slovenia

**Keywords:** VV-ECMO, pulmonary aspiration, refractory hypoxaemia, emergency laparotomy, anticoagulation, abdominal trauma, ARDS

## Abstract

**Background:** Pulmonary aspiration during induction of anaesthesia may lead to rapidly progressive, life-threatening hypoxaemic respiratory failure. This poses a particular challenge when urgent surgery cannot be deferred. **Case Presentation:** A 33-year-old man required urgent relaparotomy for mesenteric venous thrombosis and multifocal bowel gangrene after blunt abdominal trauma. During rapid-sequence induction, he aspirated approximately 230 mL of mixed liquid gastric contents and solid food particles. Despite immediate suctioning, three therapeutic bronchoscopies, volume-controlled ventilation with FiO_2_ 1.0 and PEEP 10 cmH_2_O, stepwise PEEP titration, and inhaled nitric oxide, hypoxaemia progressed. At the final pre-ECMO assessment, SpO_2_ was 68%, pH 7.329, PaO_2_ 5.1 kPa, PaCO_2_ 6.66 kPa, and the PaO_2_/FiO_2_ ratio approximately 38 mmHg. Tidal volume was 590 mL (8.0 mL/kg of predicted body weight). Plateau pressure was not measured; driving pressure and pre-ECMO static compliance were therefore unavailable. Severe refractory hypoxaemia persisted for approximately 60 min. The decision to initiate VV-ECMO was made 45 min after aspiration; as a result, full femoro-jugular flow was achieved at 105 min after aspiration, and surgical incision followed 45 min later. The operation lasted 165 min. The estimated blood loss was 500 mL, and three units of packed red blood cells were transfused without uncontrolled surgical haemorrhage. The ECMO course was complicated by excessive advancement of the right internal jugular return cannula with clinically suspected recirculation and by marked transient thrombocytopenia. VV-ECMO was discontinued on ICU day 7, and final extubation occurred on ICU day 21. The patient was discharged home after regaining independence in basic activities of daily living, with an end-ileostomy and end-descending colostomy. Intestinal continuity was successfully restored at approximately 18 months. No formal pulmonary-function or neurocognitive testing was performed during follow-up. **Conclusions:** In this patient, rescue VV-ECMO provided gas-exchange support, enabling non-deferrable abdominal surgery to proceed. However, a single case cannot establish general feasibility, safety, effectiveness, optimal timing, or patient-selection criteria.

## 1. Introduction

Perioperative pulmonary aspiration is uncommon but potentially catastrophic, and the risk is increased during emergency surgery. Large-volume aspiration can cause mechanical airway obstruction, chemical pneumonitis, secondary pulmonary infection, and acute respiratory distress syndrome (ARDS) [1,2,3,4,5]. In selected patients with acute, potentially reversible respiratory failure and refractory hypoxaemia, veno-venous extracorporeal membrane oxygenation (VV-ECMO) may provide temporary extracorporeal gas exchange [6,7].

The published literature dealing with ECMO after peri-induction or peri-procedural aspiration is limited to individual case reports and one small case series. In these reports, the planned operation was cancelled, ECMO was initiated during an ongoing operation, or extracorporeal support was instituted only after the procedure was complete [8,9,10,11,12]. Detailed reporting is particularly limited when aspiration-induced respiratory failure coincides with an abdominal condition requiring immediate surgical source control: delaying surgery risks progression of bowel necrosis, whereas ECMO cannulation and systemic anticoagulation introduce additional haemorrhagic and technical risks.

This report provides a case-based analysis of the multidisciplinary decision-making and perioperative management of rescue VV-ECMO in a patient with aspiration-induced refractory hypoxaemia requiring non-deferrable abdominal surgery. Particular attention is given to pre-ECMO physiological severity and candidacy assessment, perioperative anticoagulation and haemostasis, cannula-related complications, and documented follow-up. The observations are intended to clarify the clinical reasoning and management challenges in this rare scenario, not to establish treatment efficacy, general feasibility or safety, optimal timing, or universal patient-selection criteria. The report was prepared in accordance with the CARE reporting guidelines [13]; unavailable retrospective information is explicitly identified, and the completed checklist is provided as Supplementary File S1.

## 2. Detailed Case Description

### 2.1. Initial Trauma, First Operation, and Postoperative Deterioration

A 33-year-old man was admitted after a head-on motor-vehicle collision. He was alert (Glasgow Coma Scale 15), with a blood pressure of 107/70 mmHg and heart rate of 93 beats/min. Apart from abdominal tenderness and superficial abrasions over the pelvic region, the initial trauma survey identified no additional abnormalities. Initial haemoglobin was 145 g/L, platelet count was 250 × 10^9^/L, and international normalised ratio was 1.0. Retrospective injury coding yielded a maximum Abbreviated Injury Scale score of three for the abdominal/pelvic-contents region and an Injury Severity Score of 10.

His medical history included a previously diagnosed bicuspid aortic valve. Focused transthoracic echocardiography showed discordant aortic-stenosis parameters: peak transvalvular velocity was 3.3 m/s, mean gradient was 31 mmHg, and the calculated valve area was 0.9 cm^2^. Severe concentric left-ventricular hypertrophy and mild diastolic dysfunction were present, with preserved left-ventricular ejection fraction (71%). The valve abnormality did not result in a documented perioperative haemodynamic compromise or modify the urgency or conduct of abdominal surgery.

Contrast-enhanced abdominal CT showed approximately 400–500 mL of free intraperitoneal fluid. On delayed images, increasing attenuation of fluid adjacent to bowel loops in the left lower quadrant suggested slow venous bleeding related to bowel or mesenteric contusion. A focal aneurysmal or pseudoaneurysmal abnormality of the superior mesenteric vein was also described, together with a possible splenic contusion without definite active splenic bleeding. Emergency laparotomy identified three small-bowel mesenteric defects, two mesenteric venous bleeding points, and one partial-thickness serosal injury of the small intestine. The mesenteric defects were sutured, haemostasis was achieved, and the serosal injury was oversewn. No full-thickness perforation, macroscopic bowel devascularisation, or necrosis was documented during the first operation. One prophylactic dose of low-molecular-weight heparin was administered on postoperative day 2.

**Figure 1:**
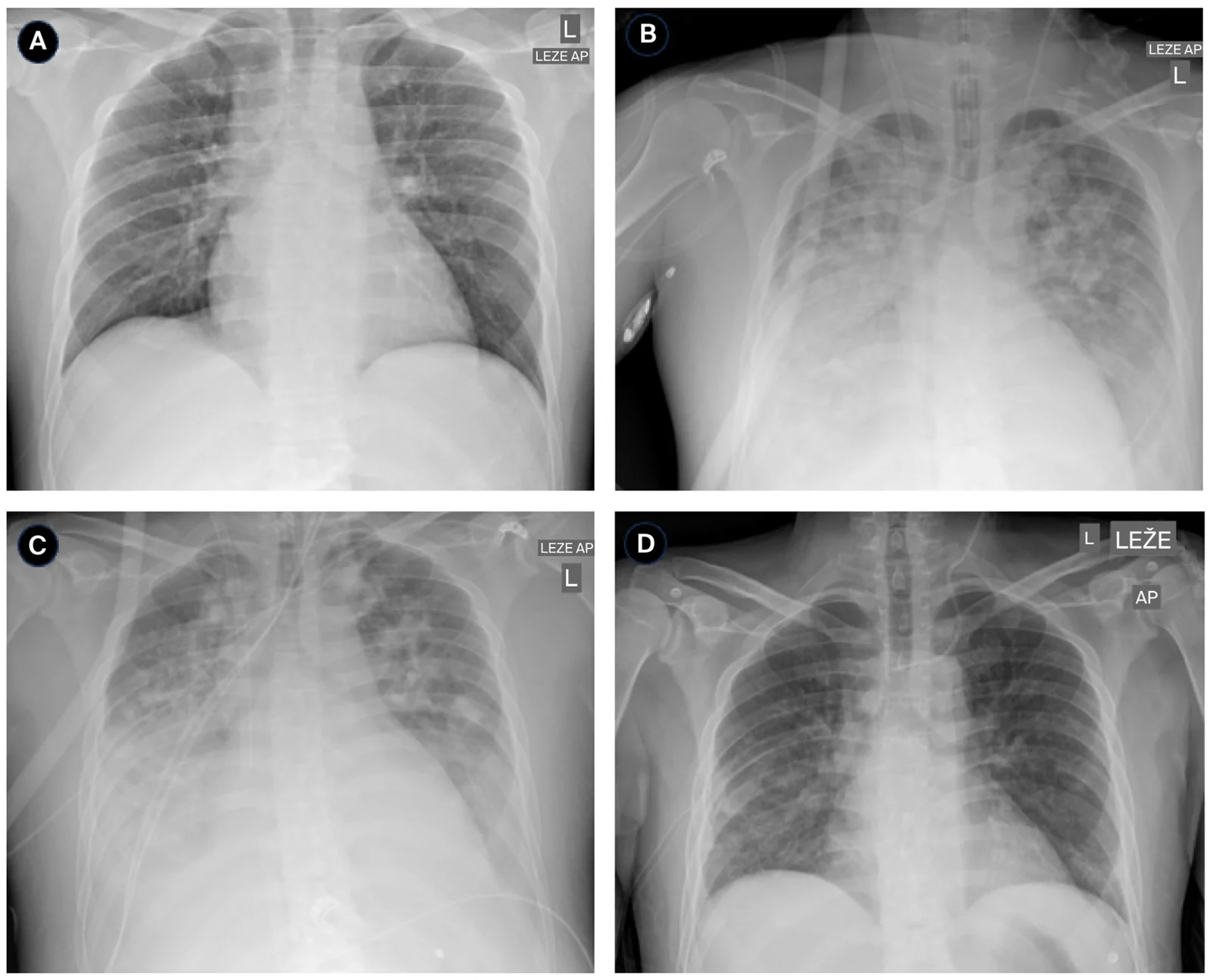
Serial chest radiographs. (**A**) Pre-relaparotomy radiograph obtained before aspiration, showing no acute pulmonary abnormality. (**B**) Intraoperative radiograph obtained after VV-ECMO initiation, showing diffuse bilateral pulmonary opacities and initial cannula malposition. (**C**) ICU radiograph obtained after cannula repositioning, showing persistent diffuse bilateral pulmonary opacities. (**D**) Post-decannulation radiograph showing marked interval improvement in bilateral pulmonary opacities. “LEŽE AP” denotes a supine anteroposterior radiograph; “L” indicates the patient’s left side.

During the first two postoperative days, the patient remained haemodynamically stable with no new abdominal or respiratory deterioration. A pre-relaparotomy chest radiograph showed no acute pulmonary abnormality and served as the pre-aspiration baseline (Figure 1A). On postoperative day 3, abrupt severe abdominal pain developed. Repeat CT demonstrated mesenteric venous thrombosis, extensive small-bowel ischaemia, small-bowel ileus with loops dilated to approximately 4.5 cm and marked gastric distension with an air–fluid level. Emergency relaparotomy was indicated.

### 2.2. Pre-Anaesthesia Status, Aspiration, and Airway Management

Immediately before emergency relaparotomy, the patient was alert (GCS 15), with a blood pressure of 130/70 mmHg, sinus rhythm of 100 beats/min, respiratory rate of 20 breaths/min, SpO_2_ 97% on room air, and temperature of 37.5 °C. Laboratory values were white-cell count: 14.2 × 10^9^/L; C-reactive protein: 399 mg/L; procalcitonin: 1.69 µg/L; haemoglobin: 97 g/L; platelet count: 130 × 10^9^/L; lactate: 1.2 mmol/L; creatinine: 98 µmol/L; and INR: 1.05.

The last documented oral intake was approximately 12 h before induction; its type and volume were not recorded. Airway assessment was Mallampati class I. Because of the emergency indication, CT-confirmed ileus, marked gastric distension with an air–fluid level, and recent abdominal surgery, the anaesthesia team considered the patient to be at high risk of aspiration and recommended nasogastric decompression. The patient remained alert but declined tube placement. The retained gastric volume was not quantified.

After head-up preoxygenation, rapid-sequence induction of anaesthesia was performed with fentanyl 200 µg, propofol 200 mg, and rocuronium 100 mg; cricoid pressure was not applied. Immediately after induction of anaesthesia and before tracheal intubation, the patient regurgitated and aspirated approximately 230 mL of mixed gastric contents comprising liquid and visible solid food particles; aspirate pH was not measured. Peripheral oxygen saturation fell abruptly after regurgitation. Immediate oropharyngeal suctioning was performed, followed by first-attempt tracheal intubation with a Cormack–Lehane grade I view. The exact interval from regurgitation to cuff inflation was not recorded. During the most severe hypoxaemia, the pupils remained equal, round, and reactive to light, and the Patient State Index was approximately 30.

After intubation, bronchoscopy revealed abundant gastric material in both main bronchi. Three therapeutic bronchoscopic airway-clearance procedures involving suction and lavage were performed before VV-ECMO cannulation; the total lavage volume and physiological response to each procedure were not recorded. No bronchospasm, airway bleeding, clinically apparent mucosal injury, or procedure-related haemodynamic instability was documented. No chest imaging was obtained between aspiration and initiation of VV-ECMO.

### 2.3. Mechanical Ventilation, Rescue Measures, and VV-ECMO Initiation in the Operating Room

At the final documented pre-ECMO assessment, volume-controlled ventilation was delivered with 1.0 FiO_2_, 10 cmH_2_O PEEP, 20 ppm inhaled nitric oxide (iNO), and a tidal volume of 590 mL (8.0 mL/kg for a predicted body weight of 73 kg). Peak airway pressure, respiratory rate, and minute ventilation were not documented. Plateau pressure was not measured; consequently, driving pressure and pre-ECMO static respiratory-system compliance could not be calculated.

With T0 defined as the time of aspiration, PaO_2_ at T0 + 15 min was 9.1 kPa on FiO_2_ 1.0, corresponding to a PaO_2_/FiO_2_ ratio of approximately 68 mmHg. At T0 + 75 min, immediately before VV-ECMO cannulation, SpO_2_ was 68%, pH 7.329, PaCO_2_ 6.66 kPa, PaO_2_ 5.1 kPa, and PaO_2_/FiO_2_ 38 mmHg (Table 1). Continuous pulse oximetry and the anaesthesia record documented approximately 60 min of progressive, refractory severe hypoxaemia before cannulation, with a nadir SpO_2_ of 68%. A stepwise PEEP titration did not produce a sustained improvement, and PEEP was subsequently maintained at 10 cmH_2_O. Prone positioning was not performed because the non-deferrable laparotomy required supine surgical access.

Anaesthesia was initially maintained with sevoflurane 2.0 vol%. An additional 50 mg of rocuronium was administered approximately 1, 2, and 3.5 h after induction. After the initiation of ECMO, sevoflurane was replaced by propofol at 5 mg/kg/h. Additional 200 µg boluses of fentanyl were administered approximately 0.5, 2, 3.5, and 5 h after induction.

Transoesophageal echocardiography (TEE) at T0 + 25 min showed hyperdynamic biventricular systolic function without right-ventricular strain or dysfunction. The multidisciplinary decision to initiate VV-ECMO and activate the ECMO team was made at T0 + 45 min. At T0 + 75 min, percutaneous ultrasound-guided access to the right femoral and right internal jugular veins was obtained and cannulation commenced. A 25-Fr/50-cm HLS venous cannula (Maquet Cardiopulmonary GmbH, Rastatt, Germany) was used for femoral drainage, and a 23-Fr/20-cm cannula was used for jugular return, as documented in the perfusion record. Before cannulation, platelet count was 130 × 10^9^/L, INR 1.05, and aPTT 32 s; baseline ACT, anti-Xa activity, fibrinogen, and viscoelastic testing were unavailable. A 5000 IU UFH bolus (approximately 50.5 IU/kg of actual body weight) was administered immediately before cannula placement.

Full VV-ECMO flow was achieved at T0 + 105 min, 60 min after the decision was made and 30 min after cannulation began. Initial circuit settings comprised a pump speed of 3000 rpm, blood flow of 4.32 L/min, sweep gas flow of 4 L/min, and FdO_2_ 1.0; circuit venous-probe saturation was 71.6%, and the oxygenator pressure gradient was 23 mmHg. Drainage- and return-line pressures as well as simultaneous pre- and post-oxygenator blood gases were unavailable. After full flow was established, SpO_2_ increased from 68% to approximately 95%, and PaO_2_ increased from 5.1 to 10.2 kPa, permitting the surgery to proceed, although ventilator FiO_2_ 1.0 and iNO 20 ppm remained necessary. Lactate increased from 2.7 mmol/L immediately before cannulation to 6.3 mmol/L immediately after full flow; no sustained hypotension was documented, and mean arterial pressure remained above 65 mmHg during this interval.

Abnormal initial cannula positioning was already apparent on the intraoperative chest radiograph (Figure 1B). Because circuit flow was stable and urgent bowel resection was prioritised, cannula adjustment was deferred until ICU admission. Given the abnormal cannula position and the persistent requirement for ventilator FiO_2_ 1.0 and iNO 20 ppm, recirculation was clinically suspected but not quantified; simultaneous pre- and post-oxygenator and independent mixed-venous oxygen measurements were not performed. Surgical incision followed at T0 + 150 min, 45 min after full VV-ECMO flow.

### 2.4. VV-ECMO-Supported Surgery and Abdominal Source Control

Relaparotomy demonstrated multifocal bowel infarction. Full-thickness transmural necrosis without macroscopic perforation involved approximately 60 cm of the terminal ileum, the caecum and proximal ascending colon, and the entire sigmoid colon from the descending–sigmoid junction to the rectosigmoid junction. All macroscopically nonviable bowel was resected. Given the multifocal necrosis, the patient’s critical condition, systemic anticoagulation, and VV-ECMO support, primary anastomosis was deferred; end-ileostomy and an end-descending colostomy were performed. The results from intraoperative abdominal samples became available later and yielded *Escherichia coli*, *Enterococcus faecalis*, *Bacteroides vulgatus*, *B. thetaiotaomicron*, *B. uniformis*, *B. ovatus*, and *Collinsella aerofaciens*; histopathological examination confirmed bowel gangrene.

The operation was completed at T0 + 315 min and lasted 165 min. Estimated blood loss was 500 mL. Intraoperative administration comprised 5000 mL of crystalloid and three units of packed red blood cells; no plasma, platelets, fibrinogen concentrate, cryoprecipitate, or other blood products were administered. Urine output was 450 mL. Haemoglobin declined from 97 g/L to a nadir of 80 g/L intraoperatively; after transfusion of three units of packed red blood cells, Hb upon ICU admission was 123 g/L. ROTEM did not identify a coagulation abnormality requiring targeted haemostatic treatment. No pump stoppage, air entrainment, oxygenator failure, circuit thrombosis, circuit exchange, or flow-limiting alarm was documented during surgery.

Approximately 2.5 h after induction of anaesthesia, hypotension required vasopressor support. Norepinephrine was initiated and reached a maximum dose of 0.42 µg/kg/min; 100 mg of hydrocortisone was administered intravenously.

### 2.5. ICU Admission and Cannula-Related Complication

The patient was admitted to the ICU approximately 6 h after aspiration. TEE demonstrated that the right internal jugular return cannula traversed the tricuspid valve and terminated in the mid-right ventricle; therefore, it was positioned too deeply. The distal tip of the long multiperforated femoral drainage cannula extended cranially to the entrance of the superior vena cava. Because drainage occurred through an extended side-hole segment, its functional position could not be inferred from the distal-tip location alone. After cannula adjustment in the ICU, comparable systemic oxygenation was maintained while ventilator FiO_2_ and iNO were reduced to 0.4 and 5 ppm, respectively, and subsequently discontinued (Table 1). The ICU chest radiograph obtained after cannula repositioning showed persistent diffuse bilateral opacities involving all four lung quadrants and the repositioned cannulae (Figure 1C).

### 2.6. Septic Shock and Microbiology

Upon ICU admission, the patient remained vasopressor-dependent due to suspected sepsis; the retrospectively calculated admission SOFA score was 11. During the first 6 h, crystalloid was administered at 200 mL/h. Additional fluid administration was guided by mini-fluid challenges, with fluid responsiveness assessed by the change in the native left-ventricular outflow-tract velocity–time integral. This was measured by focused echocardiography immediately before and after a rapid crystalloid bolus and from the contemporaneous change in ECMO blood flow. Systemic perfusion was assessed using serial arterial lactate kinetics, hourly urine output, central venous oxygen saturation, and clinical evaluation of peripheral microvascular perfusion. No monitoring of calibrated cardiac output or systemic vascular resistance was performed. Mean arterial pressure was targeted at 65 mmHg. Norepinephrine reached 0.42 µg/kg/min and vasopressin at 0.03 units/min, corresponding to a norepinephrine-equivalent dose of approximately 0.50 µg/kg/min, calculated as norepinephrine (µg/kg/min) + 2.5 × vasopressin (units/min). Both vasopressors were progressively reduced and discontinued on ICU day 2; the crystalloid infusion was reduced to 100 mL/h. Cumulative fluid balance during the first two ICU days was +7200 mL.

Bronchoalveolar lavage obtained at ICU admission was judged suitable for qualitative microbiological analysis and yielded *Klebsiella pneumoniae* and *Candida albicans*; bacterial burden was not quantified. A repeat bronchial aspirate yielded *K. pneumoniae*, *Enterococcus faecium*, and *C. albicans*. Two blood-culture sets, each containing aerobic and anaerobic bottles, were obtained approximately 12 h after ICU admission: one from the central venous catheter and one from the arterial catheter. Both yielded *K. pneumoniae* and *E. faecium*. Follow-up blood cultures were obtained approximately every 72 h and remained sterile. Empirical antimicrobial therapy comprised imipenem/cilastatin 1000 mg/1000 mg intravenously every 8 h and vancomycin, administered as a 2 g loading dose followed by 3–5 g/day in three divided doses adjusted to serum concentrations and renal function. Both antibacterial agents were administered for 10 days. Anidulafungin was started empirically and discontinued after a negative beta-D-glucan result.

An adjunctive haemoadsorption cartridge (CytoSorb^®^, CytoSorbents Corporation, Princeton, NJ, USA) was incorporated into the ECMO circuit 6 h after ICU admission. IL-6 was 137,935 ng/L at ICU admission. A second sample, obtained immediately before haemoadsorption at 6 h after ICU admission, later returned an IL-6 concentration of 23,892 ng/L; the result was not available when treatment commenced. At 18 h after ICU admission, corresponding to 12 h after treatment initiation, IL-6 was 1112 ng/L. Over the same interval, the norepinephrine-equivalent dose decreased from 0.50 to 0.135 µg/kg/min.

### 2.7. Anticoagulation, Thrombocytopenia, and Haemorrhagic Surveillance

At ICU admission, haemostatic testing showed an INR of 1.15, fibrinogen of 5.9 g/L, an antithrombin activity of 48%, a thrombin time of 15.7 s, and D-dimer of 11,031 ng/mL fibrinogen-equivalent units (FEUs). ROTEM and multiplate findings did not prompt targeted haemostatic treatment. ACT and anti-Xa activity were not measured. Continuous UFH was started after ICU admission at 1000 IU/h, initially targeting an aPTT of 55–65 s.

The platelet count decreased from 147 × 10^9^/L at ICU admission to 45 × 10^9^/L 18 h later, corresponding to an approximately 69% decrease from the ICU-admission value. One prophylactic LMWH dose was administered on day 2 following trauma and first surgery, followed by a 5000 IU UFH bolus during ECMO cannulation the next day. The retrospectively calculated 4Ts score was two—thrombocytopenia, two points; timing, 0 points; new HIT-associated thrombosis, 0 points; and other causes, 0 points—reflecting definite alternative explanations. A rapid platelet factor 4/heparin lateral-flow immunoassay, platelet factor 4/heparin enzyme-linked immunosorbent assay, and direct and indirect immunofluorescence platelet-antibody tests were negative. No functional platelet-activation assay was performed. HIT was considered very unlikely, and UFH was continued.

Given the marked thrombocytopenia and recent extensive bowel resection, the aPTT target was reduced to 45–55 s to mitigate the risk of bleeding. The platelet count subsequently recovered without platelet transfusion or other specific treatment.

Postoperatively, abdominal-drain fluid was initially serohaemorrhagic, reaching 500–750 mL/24 h, and became serous by postoperative day 2 after relaparotomy, thereafter decreasing to 100–200 mL/24 h. Recorded ileostomy output was approximately 100–300 mL/24 h, and descending-colostomy output was 100–150 mL/24 h; blood admixture was recorded once in the colostomy output. Three additional packed red-cell units were administered during the ICU course; haemoglobin was maintained at 90–100 g/L. No bleeding-related reoperation, endoscopic or radiological haemostatic procedure, or other invasive haemostatic intervention was required. No new clinically apparent thrombotic event or ECMO circuit thrombosis was documented after ECMO initiation.

### 2.8. Recovery and Follow-Up

VV-ECMO was discontinued on ICU day 7. Ventilatory support was subsequently reduced using assisted modes, predominantly adaptive support ventilation and continuous positive airway pressure, with FiO_2_ at approximately 0.25–0.40. Detailed ventilator settings from this period were unavailable. Hyperactive delirium complicated weaning. The first extubation attempt on ICU day 17 failed, and reintubation was required the same day because delirium impaired cooperation with respiratory physiotherapy and airway management. Final extubation occurred on ICU day 21.

Tracheostomy was not performed. No subsequent ventilator-associated pneumonia was diagnosed after the initial aspiration-associated pulmonary infection. No clinically apparent critical-illness neuromyopathy was documented, although no formal strength score or electrophysiological assessment was performed. Respiratory physiotherapy and progressive mobilisation continued. A bedside radiograph obtained after ECMO decannulation showed marked interval improvement of the bilateral pulmonary opacities (Figure 1D).

The patient was transferred to a lower-acuity surgical ward on ICU day 22 and discharged home 18 days later after regaining independent mobility and independence in basic activities of daily living. He had a functioning end-ileostomy and end-descending colostomy and required ongoing stoma care. At the 15-month review, the patient was alive, living independently, and reported no persistent respiratory or neurological symptoms. No formal pulmonary-function, exercise, neurocognitive, validated functional, nutritional, or quality-of-life assessment was performed. Follow-up extended to approximately 18 months, when elective restoration of intestinal continuity and stoma reversal were performed successfully without major documented postoperative complications. A separate narrative patient-perspective statement was not obtained; therefore, this section only reports documented patient-reported symptoms and functional status.

## 3. Discussion

The defining clinical dilemma was the simultaneous need to restore gas exchange and achieve urgent abdominal source control. Delaying laparotomy risked the progression of intestinal necrosis and intra-abdominal sepsis, but proceeding required VV-ECMO and systemic anticoagulation despite recent trauma and the risk of bleeding associated with urgent bowel resection. In this patient, VV-ECMO restored oxygenation sufficiently to permit resection; however, a single case cannot establish the general feasibility or safety of this strategy.

Published experience with ECMO after peri-induction or peri-procedural aspiration remains limited and heterogeneous (Table 2). In reports by Wetsch et al. and Kim et al., the planned operations were cancelled after aspiration, and ECMO was instituted 3 h and 60 min after the event, respectively [8,9]. Träger et al. initiated VV-ECMO during an ongoing urgent laparotomy; inadequate flow and right-ventricular failure subsequently required conversion to central VA-ECMO, and the course was complicated by acute kidney injury, capillary leak, and cannula revision [10]. In the case of two patients reported by Felinski et al., surgery had already been completed before postoperative ECMO rescue [11]; by contrast, Gamberini et al. instituted VV-ECMO 6 h after aspiration during procedural sedation rather than in the setting of a concurrent non-deferrable operation [12]. The present case differs in that full VV-ECMO support was established before skin incision and was maintained throughout the urgent bowel resection. This timing is clinically informative because it illustrates how competing respiratory and surgical priorities were managed; however, it should not be interpreted as evidence of a superior or optimal strategy.

The delayed mesenteric venous thrombosis was plausibly related to the documented mesenteric and superior mesenteric venous injuries. Traumatic endothelial disruption, postoperative venous stasis, and trauma-associated hypercoagulability provide a coherent mechanism for delayed thrombosis and bowel infarction; however, evolving mesenteric devascularisation that was not macroscopically apparent at the first operation cannot be excluded [14,15].

The aspiration event occurred despite an approximately 12 h interval since oral intake. The more relevant risk factors were CT-confirmed ileus and marked gastric distension, which can result in substantial retained gastric contents despite conventional fasting. The absence of pre-induction gastric decompression, therefore, resulted in a persistent anatomical substrate for regurgitation [2,3,4,5].

The subsequent respiratory syndrome was consistent with aspiration-related ARDS. Acute severe hypoxaemia followed an observed pulmonary insult, and bilateral opacities were documented on the intraoperative radiograph obtained after VV-ECMO initiation. Transthoracic and transoesophageal echocardiography showed preserved or hyperdynamic biventricular systolic function, making hydrostatic oedema from primary ventricular failure unlikely; however, perioperative fluid loading and sepsis-related capillary leak probably contributed to the radiographic pulmonary oedema. Once bilateral opacities were documented, the overall clinical course fulfilled the Berlin definition of ARDS; the pre-ECMO PaO_2_/FiO_2_ ratio of approximately 38 mmHg on 10 cmH_2_O PEEP was within the severe oxygenation range [16].

VV-ECMO candidacy was grounded in the 2021 ELSO framework for acute, potentially reversible hypoxaemic respiratory failure refractory to maximal feasible conventional support. Aspiration is listed among the relevant clinical conditions, and a PaO_2_/FiO_2_ ratio below 80 mmHg is a recognised hypoxaemic threshold [7]. In this patient, the ratio was approximately 38 mmHg despite 1.0 FiO_2_, 10 cmH_2_O PEEP, repeated bronchoscopic clearance, stepwise PEEP titration, and iNO. Prone positioning was not feasible because immediate laparotomy required supine access. Plateau pressure was not measured, and driving pressure and pre-ECMO static compliance could not be calculated. The ELSO plateau-pressure requirement applies to the separate hypercapnic indication; therefore, the missing mechanical measurements limited retrospective assessment of ventilatory stress but did not negate candidacy based on profound refractory hypoxaemia.

Respiratory rescue measures and ECMO preparation were undertaken in parallel. Three bronchoscopic suction-and-lavage procedures removed visible gastric material but did not produce sustained improvement; stepwise PEEP titration and iNO were also used while the ECMO team was mobilised. With T0 defined as the time of aspiration, the decision to initiate VV-ECMO was made at T0 + 45 min; cannulation began at T0 + 75 min; full flow was achieved at T0 + 105 min; and surgical incision followed at T0 + 150 min, 45 min after full flow. This sequence limited further delay once extracorporeal support was selected, although a single case cannot define optimal timing. The transient lactate increase from 2.7 to 6.3 mmol/L occurred without sustained hypotension or ventricular dysfunction. In the context of preceding profound hypoxaemia and ongoing mesenteric ischaemia, it was likely multifactorial, with possible contributions from accumulated oxygen debt, adrenergic stress, and reperfusion-associated washout. Lactate decreased on subsequent measurements.

The anticoagulation strategy reflected competing thrombotic and haemorrhagic risks. The 5000 IU UFH bolus corresponded to approximately 50.5 IU/kg actual body weight; it was administered to a patient with recent trauma, recent laparotomy, and known mesenteric venous thrombosis but no uncontrolled active bleeding [17,18]. The 165 min operation was associated with an estimated blood loss of 500 mL and transfusion of three units of packed red blood cells; no other blood products or invasive haemostatic intervention were required. These data document completion of the procedure without uncontrolled surgical haemorrhage but do not establish the safety of operating with ECMO. During the subsequent ECMO course in ICU, the transfusion and intervention profile did not meet the retrospectively applied ELSO Registry definition of haemorrhagic complications [19].

The early platelet decrease in ICU was more consistent with a multifactorial perioperative and extracorporeal process than with HIT. Major surgery, haemodilution and transfusion, sepsis, and ECMO-associated platelet activation and consumption were competing explanations. The initial exposure to heparin occurred on day 2 following trauma and first surgery, and the fall in platelet count began within four days without separate heparin exposure during the preceding 100 days; this yielded 0 timing points in the 4Ts calculation. Mesenteric venous thrombosis preceded both the marked platelet decline and the UFH bolus, anatomically related to the traumatic mesenteric venous lesion, so it was not classified as a new HIT-associated thrombosis. The resulting 4Ts score was two, and negative platelet factor 4/heparin immunoassays made HIT very unlikely [20]. The aPTT target was reduced to mitigate haemorrhagic risk during marked thrombocytopenia.

Cannula malposition was the principal ECMO-related mechanical complication. The right internal jugular return cannula crossed the tricuspid valve and terminated in the mid-right ventricle. Interpretation of the femoral drainage cannula was less direct because its extended multiperforated segment, rather than the distal tip alone, determined the functional drainage region. Recirculation was suspected based on the cannula geometry and persistent requirement for ventilator FiO_2_ 1.0 and iNO despite stable circuit flow; however, no recirculation fraction was measured. After repositioning, comparable systemic oxygenation was maintained using FiO_2_ 0.4 and the discontinuation of iNO. This temporal response is compatible with improved effective extracorporeal support, but neither quantifies recirculation nor establishes repositioning as the sole cause [21]. Deferring adjustment until ICU admission reflected a case-specific decision to prioritise urgent abdominal source control once oxygenation improved sufficiently to proceed.

Operative findings of multifocal bowel gangrene, histopathological confirmation, and polymicrobial intra-abdominal cultures established a complicated intra-abdominal infectious source. The immediate respiratory syndrome was most consistent with chemical aspiration pneumonitis, with an additional obstructive component from aspirated particulate material; subsequent lower-airway and blood cultures made superimposed bacterial pulmonary infection plausible but did not establish a pulmonary source as the principal driver of bloodstream infection or shock. The vasopressor-dependent state that began intraoperatively and persisted upon ICU admission was clinically consistent with septic shock in the setting of gangrenous bowel, polymicrobial intra-abdominal infection, and subsequent bacteraemia. Resection of all nonviable bowel constituted the principal source-control intervention [22,23]. Haemoadsorption was used as an adjunct during the early shock state; however, IL-6 had already decreased by approximately 83% before treatment initiation. Because surgical source control, antimicrobial therapy, hydrocortisone, fluid and vasopressor treatment, VV-ECMO optimisation, and the natural evolution of shock occurred concurrently, neither the subsequent decline in inflammatory biomarkers nor the reduction in vasopressor requirement can be attributed to haemoadsorption. These observations are hypothesis-generating only and are consistent with the uncertain clinical efficacy reported in the systematic review [24].

The documented outcome extends beyond survival to hospital discharge but does not support a claim of recovery without impairment. The patient ended VV-ECMO treatment and mechanical ventilation and returned to independent living, with no reported persistent respiratory or neurological symptoms. Long-term respiratory, physical, and neurocognitive sequelae need to be recognised after ARDS [25]. In this patient, however, formal pulmonary, neurocognitive, functional, nutritional, and quality-of-life assessments were not performed. Two stomas represented a substantial gastrointestinal consequence until intestinal continuity could be restored at approximately 18 months. The outcome is therefore best described as survival with recovery to independent daily functioning rather than a documented absence of sequelae [25].

### Limitations

This report is limited by its retrospective single-case design and incomplete physiological reconstruction. Pre-ECMO peak and plateau pressures, driving pressure, static compliance, respiratory rate, and minute ventilation were unavailable; responses to individual bronchoscopies and PEEP steps were not recorded; and pre- and post-oxygenator gases, line pressures, and quantitative recirculation measurements were not obtained. The ICU-admission SOFA score was reconstructed retrospectively; its respiratory and neurological components were influenced by active VV-ECMO support and sedation. A formal patient-perspective narrative, pulmonary-function testing, neurocognitive assessment, standardised nutritional evaluation, and validated functional or quality-of-life measures were unavailable. The numerous simultaneous interventions preclude causal attribution, and the observations cannot define general patient selection, timing, anticoagulation, or cannula-management strategies.

## 4. Conclusions

This report describes the use of rescue VV-ECMO after aspiration was observed during induction for non-deferrable abdominal surgery. In this patient, extracorporeal support restored sufficient gas exchange to permit resection of the gangrenous bowel. The course also illustrates the perioperative haemorrhagic risk and the potential for cannula-related mechanical complications. These case-specific observations do not establish general feasibility, safety, effectiveness, optimal timing, or patient-selection criteria.

## Figures and Tables

**Table 1:** Serial respiratory, arterial blood gas, and VV-ECMO data from aspiration through to early ICU course.

Parameter	T0 (Aspiration) + 15 min	T0 + 75 min (Final Pre-ECMO)	T0 + 105 min (Full Flow)	T0 + 6 h	T0 + 24 h
Event	After first bronchoscopy	After additional bronchoscopies + PEEP-increase trial + iNO	Immediately after full ECMO flow	After cannula adjustment in ICU	18 h after ICU admission
FiO_2_/PEEP	1.0/10 cmH_2_O	1.0/10 cmH_2_O	1.0/10 cmH_2_O	0.4/10 cmH_2_O	0.4/10 cmH_2_O
VT/PBW	Not documented	8.0 mL/kg	8.0 mL/kg	3.0 mL/kg	3.0 mL/kg
iNO	0	20 ppm	20 ppm	5 ppm	0
ECMO blood flow/sweep gas flow/FdO_2_	N/A	N/A	4.32 L/min/4 L/min/1.0	3.3 L/min/3 L/min/1.0	4 L/min/3 L/min/1.0
SpO_2_	91%	68%	95%	95%	98%
pH	7.295	7.329	7.341	7.364	7.401
PaCO_2_	8.51 kPa	6.66 kPa	4.53 kPa	4.42 kPa	5.27 kPa
PaO_2_	9.1 kPa	5.1 kPa	10.2 kPa	9.9 kPa	12.3 kPa
PaO_2_/FiO_2_	≈68 mmHg	≈38 mmHg	Not interpreted during ECMO	Not interpreted during ECMO	Not interpreted during ECMO
Lactate	1.0 mmol/L	2.7 mmol/L	6.3 mmol/L	5.7 mmol/L	3.6 mmol/L

Notes: T0 denotes the time of aspiration. Pre-ECMO peak airway pressure, respiratory rate, and minute ventilation were not documented; plateau pressure was not measured; driving pressure and pre-ECMO static compliance were not calculable. Post-ECMO PaO_2_/FiO_2_ values were not used to grade native-lung severity because systemic arterial oxygenation was influenced by extracorporeal support. Abbreviations: ICU, intensive care unit; SpO_2_, peripheral oxygen saturation; PaCO_2_, arterial partial pressure of carbon dioxide; PaO_2_, arterial partial pressure of oxygen; FiO_2_, fraction of inspired oxygen; PEEP, positive end-expiratory pressure; VT, tidal volume; ECMO, extracorporeal membrane oxygenation; FdO_2_, fraction of oxygen delivered through the ECMO gas circuit; iNO, inhaled nitric oxide; ppm, parts per million; PBW, predicted body weight; N/A, not applicable.

**Table 2:** Published reports of ECMO for aspiration-related respiratory failure in perioperative or peri-procedural settings, with comparison to the present case.

Study	Patient Age	Aspiration Context	ECMO Timing	Surgery Decision	Complications
Wetsch 2012 [8]	39	Induction for urgent hand surgery	VA-ECMO 3 h after aspiration; VV-ECMO later on day 7	Cancelled	Cardiac arrest before ECMO; limited oxygenation during VA-ECMO was attributed to high cardiac output, requiring subsequent VV-ECMO.
Kim 2015 [9]	64	Induction for diagnostic laparoscopy/possible gastrectomy	ECMO 60 min after aspiration	Cancelled	No ECMO-related complication was described in the report.
Träger 2016 [10]	45	Induction for urgent laparotomy for small-bowel obstruction due to torsion	VV-ECMO initiated during ongoing surgery after severe respiratory failure; converted to central VA-ECMO 4 h later because of insufficient VV-ECMO flow and right-ventricular failure	Proceeded; laparotomy performed and small-bowel decompression achieved	Right-ventricular failure requiring conversion from VV-ECMO to VA-ECMO; AKI requiring CRRT; severe capillary leak/MOF; surgical revision for arterial ECMO cannula repositioning.
Felinski 2021 [11]	Two patients, both 56	Induction for reoperation after Roux-en-Y gastric bypass surgery	Postoperative; within several hours in both cases	Completed before ECMO	Incidental subsegmental pulmonary embolism.
Gamberini 2021 [12]	69	Sedation for ERCP on postoperative day 9 after abdominal surgery	6 h after aspiration	N/A (ERCP)	Renal failure requiring haemodialysis.
Present case	33	Induction before urgent relaparotomy	Full VV-ECMO flow achieved 105 min after aspiration and 45 min before surgical incision	Proceeded under VV-ECMO support	Excessively deep right internal jugular return cannula with clinically suspected recirculation; marked transient thrombocytopenia; no ECMO circuit failure or circuit thrombosis; no ELSO Registry-defined haemorrhagic complication.

Legend: Cases were selected because aspiration was temporally related to induction of anaesthesia or procedural sedation and ECMO was required for aspiration-related respiratory failure. Abbreviations: AKI, acute kidney injury; CRRT, continuous renal replacement therapy; ECMO, extracorporeal membrane oxygenation; VA-ECMO, veno-arterial extracorporeal membrane oxygenation; VV-ECMO, veno-venous extracorporeal membrane oxygenation; ELSO, Extracorporeal Life Support Organization; ERCP, endoscopic retrograde cholangiopancreatography; MOF, multiple organ failure; N/A, not applicable.

## Data Availability

All data relevant to this case report are included in the article; additional de-identified source data are not publicly available because of patient confidentiality.

## Supplementary Materials

The following supporting information can be downloaded at: https://www.mdpi.com/article/10.3390/jcm15176746/s1, File S1: CARE Reporting Checklist and Clinical Timeline.

## Abbreviations

The following abbreviations are used in this manuscript:

ACT	activated clotting time
AKI	acute kidney injury
aPTT	activated partial thromboplastin time
ARDS	acute respiratory distress syndrome
CRRT	continuous renal replacement therapy
CT	computed tomography
ECMO	extracorporeal membrane oxygenation
ELSO	Extracorporeal Life Support Organization
ERCP	endoscopic retrograde cholangiopancreatography
FdO_2_	fraction of delivered oxygen via ECMO
FiO_2_	fraction of inspired oxygen
GCS	Glasgow Coma Scale
HIT	heparin-induced thrombocytopenia
ICU	intensive care unit
IL-6	interleukin-6
iNO	inhaled nitric oxide
INR	international normalised ratio
IU	international unit
LMWH	low-molecular-weight heparin
LVOT VTI	left-ventricular outflow-tract velocity–time integral
MAP	mean arterial pressure
MOF	multiple organ failure
N/A	not applicable
PaCO_2_	arterial partial pressure of carbon dioxide
PaO_2_	arterial partial pressure of oxygen
PaO_2_/FiO_2_	ratio of arterial oxygen tension to inspired oxygen fraction
PBW	predicted body weight
PEEP	positive end-expiratory pressure
ppm	parts per million
ROTEM	rotational thromboelastometry
SOFA	Sequential Organ Failure Assessment
SpO_2_	peripheral oxygen saturation
T0	time of aspiration
TEE	transoesophageal echocardiography
TTE	transthoracic echocardiography
UFH	unfractionated heparin
VA-ECMO	veno-arterial extracorporeal membrane oxygenation
VV-ECMO	veno-venous extracorporeal membrane oxygenation
