# Supplementary material for: Rescue Veno-Venous Extracorporeal Membrane Oxygenation for Aspiration-Induced Refractory Hypoxaemia Before Non-Deferrable Abdominal Surgery: A Case Report
Source: J Clin Med. 2026 Aug 30;15(17):6746. doi: 10.3390/jcm15176746 (PMC13565860; doi:10.3390/jcm15176746)
Supplement: Supplementary file 1 [file jcm-15-06746-s001.zip › jcm-4468666-supplementary.pdf]

# Supplementary File S1 — CARE Reporting Checklist and Clinical Timeline

NOTE: Page and line references refer to the submitted 16-page line-numbered revised-manuscript PDF.

## CARE checklist

| Item | CARE domain | Current location | How addressed | Status/notes |
|---|---|---|---|---|
| 1 | Title | p. 1, lines 1–4 | The title identifies the intervention, principal clinical problem, and article type as a case report. | Reported |
| 2 | Keywords | p. 1, lines 35–37 | VV-ECMO, pulmonary aspiration, refractory hypoxaemia, emergency laparotomy, anticoagulation, abdominal trauma, and ARDS. | Reported |
| 3 | Abstract | p. 1, lines 5–34 | Background, case presentation, objective pre-ECMO severity, intervention, complications, outcome, and cautious conclusion are reported. | Reported; unsupported causal and feasibility claims removed |
| 4 | Introduction | p. 1, lines 40–42 and p. 2, lines 43–62 | Background, directly relevant aspiration-related ECMO literature, precise clinical gap, case-based aim, and limits of inference. | Reported |
| 5 | Patient information | Section 2.1, p. 2, lines 66–83 and p. 3, lines 84–93; Section 2.2, p. 3, lines 95–105 | Age, trauma presentation, relevant medical history, presenting concerns, initial laboratory values, injury severity, and pre-relaparotomy status. | Reported |
| 6 | Clinical findings | Sections 2.1–2.3, pp. 2–5, lines 67–165 | Admission findings, CT and operative anatomy, airway assessment, aspiration event, respiratory physiology, echocardiography, ECMO findings, and cannula position. | Reported; unavailable findings identified |
| 7 | Timeline | Chronological Case Description, pp. 2–9, lines 66–288; Table 1, p. 6; supplementary clinical timeline below | The narrative is chronological; Table 1 details the aspiration-to-early-ICU interval; the supplementary timeline below summarises the full episode through 18 months. | Reported |
| 8 | Diagnostic assessment | Sections 2.1–2.6, pp. 2–7, lines 66–239; Discussion, p. 11, lines 315–350 and p. 12, lines 379–393 | Traumatic mesenteric venous injury/thrombosis, aspiration-related respiratory failure and ARDS, alternative pulmonary-oedema contributors, septic sources, HIT assessment, and diagnostic uncertainty. | Reported; causal uncertainty explicit |
| 9 | Therapeutic interventions | Sections 2.2–2.7, pp. 3–8, lines 95–266; Table 1, p. 6 | Induction drugs, airway suctioning/bronchoscopy, ventilation and rescue measures, VV-ECMO configuration/settings, anticoagulation, surgery, source control, haemodynamic treatment, antimicrobials, and treatment modifications. | Reported; unavailable doses/measurements identified |
| 10 | Follow-up and outcomes | Section 2.8, p. 8, lines 268–279 and p. 9, lines 280–288 | Bleeding and thrombotic surveillance, ECMO complications, ventilator weaning, rehabilitation, discharge status, stomas, 15-month review, and restoration of continuity at approximately 18 months. | Reported; no validated long-term functional testing |
| 11 | Discussion | Discussion, p. 9, lines 290–308; Table 2, p. 10; pp. 11–13, lines 315–413 | Relevant literature, rationale, case-specific interpretation, competing explanations, strengths, limitations, and restricted conclusions. | Reported |

| Item | CARE domain | Current location | How addressed | Status/notes |
|---|---|---|---|---|
| 12 | Patient perspective | Section 2.8, p. 9, lines 283–288 | A separate narrative patient-perspective statement was not obtained. Documented patient-reported respiratory and neurological symptoms and functional status are reported. | Partially available; limitation stated transparently |
| 13 | Informed consent | Informed Consent Statement, p. 13, lines 429–430 | Written informed consent for publication of the case and accompanying images was obtained. | Reported |

# CARE-aligned clinical timeline

| Time | Clinical event | Manuscript location |
|---|---|---|
| **Trauma day 0** | Head-on motor-vehicle collision; GCS 15; haemodynamically stable; CT showed 400–500 mL intraperitoneal fluid, suspected slow venous bleeding, and superior mesenteric venous abnormality. | p. 2, lines 67–83 |
| **Trauma day 0** | Emergency laparotomy: three mesenteric defects, two mesenteric venous bleeding points, and one partial-thickness small-bowel serosal injury were treated. | p. 2, line 83 and p. 3, lines 84–87 |
| **Postoperative day 2** | One prophylactic LMWH dose administered. | p. 3, line 87 |
| **Postoperative day 3** | Abrupt severe abdominal pain; repeat CT showed mesenteric venous thrombosis, extensive bowel ischaemia, ileus, and marked gastric distension; emergency relaparotomy indicated. | p. 3, lines 88–93 |
| **T0** | T0 defined as witnessed aspiration during induction of anaesthesia before tracheal intubation. | p. 3, lines 106–118 |
| **T0 + 15 min** | $PaO_2/FiO_2$ approximately 68 mmHg after initial bronchoscopy. | p. 4, lines 131–135; Table 1, p. 6 |
| **T0 + 25 min** | TEE showed hyperdynamic biventricular systolic function without right-ventricular dysfunction. | p. 5, lines 142–143 |
| **T0 + 45 min** | Multidisciplinary decision to initiate VV-ECMO and ECMO-team activation. | p. 5, lines 143–144 |
| **T0 + 75 min** | Final pre-ECMO assessment: $SpO_2$ 68%, $PaO_2/FiO_2$ approximately 38 mmHg; cannulation commenced. | p. 4, lines 132–135 and p. 5, lines 144–150 |
| **T0 + 105 min** | Full VV-ECMO flow achieved; $SpO_2$ and $PaO_2$ improved sufficiently to proceed. | p. 5, lines 151–159 |
| **T0 + 150 min** | Surgical incision; relaparotomy confirmed multifocal transmural bowel gangrene. | p. 5, lines 164–165 and p. 6, lines 179–187 |
| **T0 + 315 min** | Operation completed after 165 min; 500 mL estimated blood loss; three packed red-cell units; no other blood products. | p. 6, lines 188–197 |
| **Approximately T0 + 6 h** | ICU admission; return cannula identified as excessively deep; cannulae repositioned; ventilator $FiO_2$ reduced subsequently. | p. 7, lines 199–208 |
| **ICU admission + 6 h** | Adjunctive haemoadsorption initiated; immediately pre-treatment IL-6 sample later measured 23,892 ng/L. | p. 7, lines 234–237 |
| **ICU admission + 18 h** | Platelet nadir 45 × $10^9$/L; IL-6 1,112 ng/L at 12 h after haemoadsorption initiation. | p. 7, lines 237–239 and p. 8, lines 247–258 |
| **ICU day 2** | Norepinephrine and vasopressin discontinued. | p. 7, lines 219–223 |
| **ICU day 7** | VV-ECMO discontinued. | p. 8, lines 268–269 |

| Time | Clinical event | Manuscript location |
|---|---|---|
| **ICU day 17** | First extubation attempt failed; reintubated the same day. | p. 8, lines 271–274 |
| **ICU day 21** | Final extubation. | p. 8, line 274 |
| **ICU day 22** | Transfer to lower-acuity surgical ward. | p. 9, line 280 |
| **18 days after ward transfer** | Discharged home independent in basic daily activities, with end ileostomy and end descending colostomy. | p. 9, lines 280–282 |
| **15-month review** | Living independently; no persistent respiratory or neurological symptoms reported; no formal pulmonary or neurocognitive testing. | p. 9, lines 283–285 |
| **Approximately 18 months** | Successful restoration of intestinal continuity and stoma reversal. | p. 9, lines 285–287 |
